# Dorsal root ganglion stimulation for patients with chronic pelvic pain: A retrospective review of patient experiences and long-term outcomes^☆^

**DOI:** 10.1016/j.inpm.2024.100397

**Published:** 2024-03-10

**Authors:** Stacey L. Burns, Petra Majdak, Alexandra R. Adler, Celine Jo, Michael C. Chiang, Robert Jason Yong, Antje M. Barreveld

**Affiliations:** a Brigham and Women's Hospital, Harvard Medical School, 75 Francis Street, Boston, MA, 02115, USA; b Newton Wellesley Hospital, 159 Well Ave, Pain Service, Newton, MA, 02459, USA; c Lowell General Hospital, 2 Hospital Dr 2nd Floor, Lowell, MA, 01852, USA; d Tufts University School of Medicine, 145 Harrison Ave, Boston, MA, 02111, USA

**Keywords:** Neuromodulation, Chronic pelvic pain, Dorsal root ganglion stimulation, Lead migration and fracture, Spinal cord stimulation

## Abstract

**Introduction:**

Chronic pelvic pain (CPP) is a refractory condition that has physical, emotional, and financial impacts on patients. Dorsal root ganglion stimulation (DRGS) is a promising interventional modality for patients with refractory CPP, however studies of long-term outcomes are limited. We aim to present the results from a retrospective review of 31 patients with CPP treated using DRGS.

**Materials and methods:**

IRB approval was obtained. A retrospective chart review was conducted, including 31 patients who underwent a DRGS trial between 2017 and 2022 at two academic centers. Pain history, trial/implant lead configuration, complications/revisions, pain scores, functional goals, and medication use were recorded.

**Results:**

Thirty-one patients with CPP underwent a 7–10 day DRGS trial between 2017 and 2022. Of the 31 patients, 21 (68%, CI 50–81%) had a successful trial, defined as >50% reported pain relief. Twenty patients underwent DRGS implantation. Average follow-up was 28.2 ± 17.3 months. Nine patients (45%) required revision surgery for lead migration or fracture. Thirteen patients remain implanted with an average reported percent relief of 55 ± 15%. Seven patients were explanted (35%), with an average time to explant of 12.5 ± 3 months.

**Conclusions:**

This study presents one of the largest groups of patients with DRGS for the treatment of CPP. The results highlight the variable experiences of patients after DRGS trial/implant. We report on the incidence of lead migration and fracture, sparingly described in the literature. Larger, prospective studies are needed to elucidate which patients with CPP may benefit most from DRGS, and to better understand the incidence and implications of complications.

## 1 Introduction

Chronic pelvic pain (CPP) is a condition of heterogenous pain syndromes that affects up to 16% of men and women globally and takes a financial and emotional toll on patients, their families, and the healthcare system [[1], [2], [3], [4]]. The American College of Obstetricians and Gynecologists (ACOG) defines CPP as pain lasting at least six months caused by pelvic organs or systems including gynecologic, urologic, gastrointestinal, musculoskeletal, neurologic, or psychiatric, of which the clinical manifestations lack clear identifiable causes [5]. While the pathophysiology of CPP is multifactorial and under active investigation [6,7], current theories posit a key role for central sensitization [5,8]. In support of this, patients commonly report neuropathic symptoms including paresthesias, hyperalgesia, and allodynia that interfere with activities of daily living and quality of life [9,10]. Neuropathic pain in CPP is typically managed with physical therapy, nerve blocks, medications ranging from anti-inflammatories to antidepressants and anticonvulsants, and surgical interventions. Despite this, many patients remain refractory to treatment.

More recently, neuromodulation via dorsal root ganglion (DRG) stimulation (DRGS) has been reported as an effective intervention for refractory CPP of various etiologies [11]. Compared to traditional spinal cord stimulation (SCS), which non-selectively targets passing fibers within the dorsal columns of the spinal cord, DRGS directly stimulates the DRG, a potential pain generator. More broadly, DRGS has been studied as a potential therapy for neuropathic chronic pain conditions such as complex regional pain syndrome, post-surgical pain, and peripheral neuropathy [12]. Some studies suggest that DRGS is an effective alternative to dorsal column SCS; in 60 patients who had chronic pain of various etiologies refractory to traditional SCS, DRGS was found to have a 90% trial-to-implantation rate, indicating responsiveness to DRGS among patients without sustained benefit from SCS [13]. Rowland et al. (2016) reported the first successful case of treatment with DRGS for CPP, in which DRG leads were implanted at L1 and L2 for a patient with severe pelvic girdle pain related to prior pregnancies [14]. This patient experienced functional improvements with sustained effect, demonstrating the potential of DRGS to ameliorate pelvic pain despite failure with more conservative therapies.

Few studies have evaluated the efficacy of DRGS implants in CPP patients. Hunter et al. (2019) first published the results of a novel lead configuration of DRGS (leads placed at the bilateral L1 and S2 DRG) for the treatment of CPP [15]. In this retrospective case series of seven patients with CPP who underwent DRGS, all patients reported an improvement in pain, a reduction in opioid consumption, and in some cases, an improvement in sexual function and urination, with no explants reported at the one-year follow-up point [15]. Hunter et al. also demonstrated that the best predictor for successful pain relief from DRGS is the extent of paresthesia coverage of the painful area during programming of the DRGS [15]. Another case series of 15 patients, published by Patel et al. (2019), found that all 15 patients with CPP who underwent DRGS implant had improvement in function, symptoms, numeric rating scale (NRS) pain score, and modified Oswestry Disability Index (ODI) at the three-month follow-up point [16]. In one prospective cohort study, 55 patients with neuropathic CPP were identified, of which 11 underwent various types of neuromodulation, while the remaining 44 were medically managed [17]. The neuromodulation patients did not have a significant improvement in overall NRS, but did have improvement in other domains including worst NRS and pain catastrophizing scores [17]. While this study is not limited specifically to DRGS for CPP, it does demonstrate the utility of neuromodulation in this notoriously refractory condition [11,17]. Currently, the best predictor for successful pain relief from DRGS is the extent of paresthesia coverage of the painful area during programming of the DRG device [15]. However, little is known about other predictors for success or failure with DRGS in patients with CPP, or whether paresthesia stimulation may lead to benefits in a subgroup of patients.

Based on the available evidence, DRGS appears to be a promising treatment modality for patients with refractory CPP. However, sample sizes and follow-up time intervals are limited, and there is a lack of information on the incidence of complications during the trial and/or implant period. Drawing from the SCS literature, despite its efficacy for several chronic pain conditions, this technology is associated with a relatively high rate of surgical revisions and explants, especially in the CPP population [18]. It logically follows that DRGS may have similar limitations, which makes the need for larger studies even more vital. DRGS has been studied in the treatment of chronic neuropathic pain in Denmark, where despite reported reductions in pain, the use of this technology was paused due to complications with maintaining and revising leads [19]. The risk of neurologic injury due to lead complications in the Danish study, in addition to high rates of lead fractures in other DRGS studies [[20], [21], [22]], prompted investigators to evaluate for improvements in procedural techniques, including an ipsilateral paramedian approach to minimize the risk of lead migration and fracture [23], as opposed to the traditional contralateral approach [19].

This study adds to the existing literature by reporting the outcomes of 31 patients with CPP-related diagnoses who underwent DRGS trial and/or implantation between 2017 and 2022 at two academic medical centers. Varying lead configurations were used based on patient pain distribution and inciting pathology. We report descriptive statistics, CPP histories and characteristics, and lead configurations chosen for trial and implant. We present the procedural logistics and functional response from the DRGS trial and permanent implant, if applicable. In addition, we provide information on the incidence of lead migration and fracture, revision surgery, reported side effects, and complications. We aim to compare our findings to those previously published, and describe patient- and/or systems-related issues and complications encountered throughout the process, in order to identify targets for future studies and quality and process improvement.

## 2 Materials and Methods

This study is a retrospective chart review of 31 patients who underwent a trial of DRGS for CPP of various etiologies between January 2017 and December 2022. The study protocol was approved by the Institutional Review Boards at Mass General Brigham (Protocol 2021P003703) and Tufts Medical Center/Lowell General Hospital (Protocol STUDY00003437) prior to patient enrollment. A Data Use Agreement (Number 2023A004230) was created to enable the sharing of data between the two institutions for the purposes of this study.

Patients were selected based on the ACOG definition of CPP [5]. Diagnoses included pudendal neuralgia/neuropathy, coccygodynia, and/or other neuropathic-pain conditions in the pelvis. Inclusion criteria included patients presenting with at least six months of persistent, intractable pain affecting activities of daily living (ambulation, bowel and bladder function, dressing, hygiene, and transfers). The reported pain was refractory to conservative, pharmacologic, cognitive, behavioral, and interventional modalities. Failed multimodal regimens included pelvic floor therapy, physical therapy, alternative therapies (acupuncture, massage), oral medications (muscle relaxants, gabapentinoids, antidepressants, opioids, rectal or vaginal suppositories), interventional procedures (peripheral nerve blocks, onabotulinumtoxin A to the pelvic floor), or in less common cases surgical interventions (e.g. pudendal nerve decompression).

Patients eligible for DRGS implantation underwent a 7–10 day DRGS trial with an FDA-approved system for DRGS (Proclaim DRG System, Abbott, USA) for CPP between January 2017 and December 2022 with a single provider within the Mass General Brigham System at Newton-Wellesley Hospital and two providers within the Tufts Medicine/Lowell General Hospital system. All procedures were performed by board-certified interventional pain management physicians with extensive experience in neuromodulation techniques. Trials were performed by Dr. Antje Barreveld (Newton-Wellesley Hospital, Newton, MA) and Drs. Alexandra Adler and Benjamin Henkle (Lowell General Hospital, Lowell, MA). Implantations were done between June 2018 and December 2022 by Dr. Robert Jason Yong (Brigham and Women's Hospital, Boston, MA) or Dr. Alexandra Adler (Lowell General Hospital). Pacific Interpreter Services was available to be utilized if needed.

The etiology of CPP was organized into three categories to encompass the diversity of medical diagnoses attributed to CPP: traumatic, surgical, or unknown (unspecified). Patients who were previously diagnosed with endometriosis were ultimately categorized under post-surgical CPP due to their history of prior surgical procedures with multiple co-existing diagnoses.

The DRGS trials were performed as outpatient procedures. Leads were placed percutaneously under fluoroscopic guidance at L1, S2 and/or S3 depending on each patient's pain distribution; this approach is based on previously published lead configurations and manufacturer recommendations [15]. The duration of the DRGS trial was 7–10 days. Following lead placement, an external pulse generator was connected to the leads and turned on to generate a paresthesia in the affected regions of pain. This process was repeated until the patient reported coverage of all painful regions. Patients who reported >50% improvement in pelvic pain intensity during the DRGS trial (which is the definition of a “successful trial” by the Centers for Medicare and Medicaid Services [24]) were offered DRGS implantation. Patients who did not report >50% improvement in pain intensity were not implanted.

DRGS implantation was performed as an outpatient or day surgery procedure with percutaneous placement of leads under fluoroscopic guidance. Paresthesia mapping was performed depending on the preference of the implanting physician. The leads were tunneled and connected to an implantable pulse generator, which was then implanted subcutaneously. Programming of the system was performed by the manufacturer representative under the guidance of the implanting physician.

Retrospective chart review utilizing the electronic medical record system (EPIC) enabled collection of demographic and clinical data for each patient. In addition to demographics, the following information was obtained from chart review: pain history (onset, location, duration, quality, severity, associated symptoms, alleviating/aggravating factors, radiation), comorbidities and pain-related diagnoses, previous interventions and responses, pre-and post-trial and/or implant pain scores if available, functional goals, trial details and reported percentage of pain relief, implant details for those who decided to pursue permanent DRGS implant, and any subsequent need for revision, re-operation or explantation.

Patients without follow-up within the past year and/or missing information were contacted for a voluntary phone interview. Interviews were conducted after obtaining verbal consent and using an IRB-approved script, with the goal of assessing outcomes and better understanding patients’ experiences with the device. Follow-up survey data reflecting patient satisfaction with DRGS implantation was obtained using a five-point Likert Scale with the following options: “very dissatisfied”, “dissatisfied”, “neither satisfied or dissatisfied”, “satisfied”, and “very satisfied”. Patients had the option to express specific positive or negative feedback. Lastly, patients were asked to quantify how much overall percent pain relief they were receiving from the stimulator at the date of last follow-up.

Both quantitative and qualitative data analyses were performed. Quantitative data were analyzed using basic functions in Microsoft Excel and JMP (Version 16) and are presented in the form of means, standard deviation (SD) of the means, 95% confidence intervals (CI), frequencies, and distributions. 95% confidence intervals were calculated using the Modified Wald method. The distribution of lead configuration in both trials and implants were recorded. The percentage of patients with successful trials (defined as >50% relief of the target pain), patients with procedural issues related to the trial/implant, and those requiring revision surgery or explant are described. Furthermore, the consistency of lead configuration between trial and implant is reported. Qualitative reasons for explant, reported percent pain relief, and overall patient experience and feedback are summarized.

## 3 **Results**

A total of 31 patients with CPP underwent a 7–10 day DRGS trial between 2017 and 2022. Table 1, Table 2 present the demographic data, pain history, and DRGS trial details for all 31 patients. The mean age was 57.1 ± 14.3 years, and most patients were female (77%) and Caucasian (97%). Patients reported CPP of various etiologies with the most common etiology being post-surgical chronic pain (58%). Other causes included trauma (16%) or unknown causes (26%). The average pain duration prior to a DRGS trial was 11 ± 5 years (Table 1). Twenty-one of 31 patients (67%) reported not being sexually active secondary to pain.

**Table 1:** Patient demographics and pain history.

ID	Age/Sex	Race	Pain Onset	Duration (Years)	Pain Description (Location, Quality, Associated Symptoms)
1	63/F	Caucasian	Traumatic vaginal delivery, worsened after subsequent hysterectomy with vaginal sling complicated by mesh erosion	35	Vaginal burning, foreign body sensation, urinary frequency, painful intercourse
2	53/F	Caucasian	Surgical, multiple vulvectomies for recurrent abscess formation	11	Vulvar left > right, perineal with radiation to left > right inner thigh, burning, allodynia, hyperesthesia, urinary frequency/incontinence, painful intercourse
3	37/F	Caucasian	Traumatic vaginal childbirth due to shoulder dystocia, vaginal laceration requiring re-repair	8	Left vaginal pain at introitus and internally throbbing, shooting, aching pain, painful intercourse
4	53/F	Caucasian	Cancer-related, started after radiation for anal cancer	8	Sacral pain near rectum, ischial bones, aching, burning, throbbing, dyschezia
5	70/F	Caucasian	Unknown (no inciting event)	12	Internal rectal, internal vaginal, burning, fullness, hot poker, painful intercourse
6	39/F	Caucasian	Surgical, worsened after hysterectomy	3	Rectal > vaginal, perineal pain, burning, urinary retention, painful intercourse
7	62/F	Caucasian	Injury/trauma, fractured sacrum after fall	7	Perianal, rectal, vulvar pain, dull, achy, burning, spasms, painful intercourse
8	57/F	Caucasian	Surgical, after hysterectomy with pelvic suspension and sling surgery	4	Right vagina, rectum, perineal, occasional vulva and inner groin pain, stabbing, burning, aching, urinary hesitancy, fecal incontinence, painful intercourse
9	47/F	Caucasian	Surgical, 2 months after vaginal hysterectomy	2	Right vagina radiating to groin/inner and anterior thigh, burning, sharp, shooting, allodynia, painful intercourse
10	83/M	Caucasian	Surgical, after bilateral inguinal hernia repair with mesh and transurethral prostate surgery	9	Right lower abdomen, groin, testicle, scrotum to penis, sharp, stabbing, allodynia, dysuria, painful intercourse
11	55/F	Caucasian	Surgical, after vulvectomy for cancer with multiple revision surgeries	7	Vulva, vagina, radiates to perineum, rectum, inner thigh, burning, stabbing
12	81/M	Caucasian	Cancer-related, after external beam radiation therapy for prostate cancer complicated by urethral stenosis	6	Entire penis > rectum, spasm, sharp, stabbing, shooting, fecal urgency and spasm, chronic urinary retention with suprapubic tube
13	56/F	Caucasian	Unknown (no inciting event)	21	Left rectum, ischial bone, gluteal region > vaginal pain, gnawing, throbbing, stabbing, allodynia, dyschezia, painful intercourse
14	34/F	Caucasian	Unknown (no inciting event), started with menses	16	Vaginal (internal and external), radiates to clitoris and suprapubic region, burning, allodynia, chronic urinary hesitancy, constipation, painful intercourse
15	58/F	Caucasian	Surgical, after ostomy reversal surgery complicated by infection	5	Rectal pain, burning, spasm, dyschezia, fecal urgency
16	54/F	Caucasian	Surgical, after myomectomy, then had hysterectomy which made pain worse	10	Left vaginal, labial, perineal, anal/rectal pain, can radiate to left posterior thigh, and suprapubic region, sharp, shooting, stabbing, dyschezia, constipation, painful intercourse, urinary hesitancy and retention
17	59/F	Caucasian	Unknown (no inciting event), possibly related to constant sitting	12	Vaginal, rectal, perineum, gluteal, stabbing, lightning bolt, squeezing, foreign body sensation, allodynia, urinary incontinence and frequency, painful intercourse
18	71/F	Caucasian	Surgical, after operation for bladder prolapse and hysterectomy	5	Rectum/anus left > right, internal vagina, left gluteal region, coccyx, sharp shooting, stabbing, saddle anesthesia, urinary frequency, dyschezia
19	75/F	Caucasian	Surgical, after hysterectomy	28	Vaginal, perineum, rectal, pressure, foreign body sensation, burning
20	41/F	African American	Unknown (no inciting event), worsened after abortion procedure	6	Deep pelvis bilaterally, suprapubic region, vagina, behind clitoris burning, soreness, cramping, pressure, painful intercourse, dysuria, urinary hesitancy
21	60/F	Caucasian	Surgical, after rectocele repair	8	Rectal right > left, coccyx, vagina, radiates to perineum, inner thigh, aching, burning, dyschezia
22	74/F	Caucasian	Injury/trauma, fall	17	Initially left vaginal, evolved to be more focused in rectum and coccyx, burning/hot, painful intercourse and intermittent bowel/bladder incontinence
23	53/F	Caucasian	Surgical, after L inguinal hernia repair with mesh, subsequent triple neurectomy and multiple mesh revisions	13	Left groin and vagina, sharp and burning
24	61/M	Caucasian	Surgical, after transurethral resection of the prostate	15	Rectal, perineal, buttock and inner thigh, burning, aching and sharp, urinary dysfunction
25	82/M	Caucasian	Prostatectomy	27	Left aspect of penis, burning and stabbing, urinary frequency (up to 10x per night)
26	36/F	Caucasian	Surgical, after total abdominal hysterectomy bilateral salpingectomy	7	Lower abdomen, pubic area and vagina, stabbing and sharp like “a bowling ball wrapped in barbed wire”, urinary urgency/frequency
27	65/M	Caucasian	Injury/trauma (fall)	9	Perineum, burning, urinary difficulties (leakage, incomplete voiding with overflow incontinence, has to self-catheterize)
28	56/M	Caucasian	Surgical, after R inguinal hernia repair	5	Right scrotum, intermittent burning
29	43/F	Caucasian	Injury/trauma, fall	6	Vaginal, clitoral and rectal, sharp and burning, urinary hesitancy/incontinence, dysfunctional defecation
30	64/F	Caucasian	Surgical, after hysterectomy	23	Vaginal and rectal, burning, aching
31	30/M	Caucasian	Unknown, no inciting event	12	Genital area and hips, aching, sharp, erectile dysfunction, slow voiding, post-void dribbling and pain worsened by bowel movements

Table 1 summarizes the electronic medical record data reviewed for 31 patients who underwent a trial and/or DRGS implant at Newton-Wellesley Hospital, Brigham and Women's Hospital, or Lowell General Hospital between 2017 and 2022, including demographic information, pain onset, duration, and descriptors related to pain experience.

**Table 2:** Summary of patient trial data.

ID	Goals of DRGS	Lead Configuration (number)	Complications	% Relief From Trial	Patient goals met?	Implanted (Yes/No)
1	Walk for 1 mile, resume sexual activity	Bilateral L1/S2 (4)	None	70	Yes, improvement in vaginal burning and able to walk at least 3 miles	No: pain improved after trial; did not pursue implant
2	50% pain relief, sit for longer, work more hours, less pain with intercourse	Left S2 (1) (insurance prohibited bilateral placement)	Decreased relief due to S2 lead migration, skin reaction to adhesive	55	Yes, was able to sit for longer periods of time, >50% reduction in average pain score	Yes
3	Less pain with sitting and intercourse	Left L1/S2 (2)	None	65	Yes, 100% relief of shock-like pain in introitus/perineum, 30–40% relief of pain with sitting/intercourse	Yes
4	Decrease daily pain, sit for 20+ minutes	Bilateral L1/S2 (4)	None	80	Yes, 100% relief of burning pain when standing, 60% relief of sitting pain, able to sit longer	Yes
5	Sit longer, decrease pain medications, less painful intercourse, bike ride, hike	Bilateral L1/S2 (4)	None	60	Yes, 100% relief of rectal pain, 65% relief of vaginal pain, decreased pain medications	Yes
6	Be able to come off disability to work, be able to sit	Bilateral S2/S3 (4)	None	65	Yes, able to sit, be active for longer, less overall pain	Yes
7	Return to work, less painful intercourse, sit longer	Bilateral S2, left L1 (3) (unable to place Right L1 lead 2/2 discomfort)	None	55	Yes, able to sit for longer, less painful intercourse	Yes
8	Be able to bend over to do household chores, sit and drive further distances, go to the grocery store	Right L1/S2 (2)	Skin reaction to Chlorhexidine	60	Yes, able to sit, less pain with climbing stairs, walked without a cane, long walks, went to the grocery store	Yes
9	More physical activity, walk longer, longer periods of household activities, walk up stairs alone	Right L1/S2 (2)	None	95	Yes, walked up stairs without assist, able to walk long distances without pain, sat and drove for 4 h	Yes
10	50% pain relief, fewer or less intense pain flares, decrease pain medications, more physical activity	Right T12/L1 (2)	None	50	Yes, overall less pain flares and relief of abdominal and groin pain but no relief in testicle pain	Yes
11	Less pain with activities around the house, be able to leave the house and drive, sit for 30 min	Bilateral L1/S2 (4)	Increased lower extremity and vulvar pain, even with reprogramming	0	No, felt increased pain, especially in lower extremities	No
12	Reduce the intensity and frequency of spasms, less pain with bowel movements, decrease pain medications	Left L1/S2 (2)	None	0	No, no relief of rectal or penile pain	No
13	Drive and sit for 35 min, be able to tolerate sitting during travel, wear underwear	Left S2/S3 (2)	None	0	No, no relief	No
14	Resume physical workouts for conditioning, resume socialization and possibly dating	Bilateral L1/S2 (4)	None	60	Yes, less overall pain, fewer episodes of clitoral stimulation, able to participate in a workout	Yes
15	Decrease pain with bowel movements, less rectal spasms and urgency, decrease pain medications, walk for longer	Right S2/S3 (2)	None	60	Yes, fewer bowel movements with less pain and spasm, able to double walking distance	Yes
16	Decrease pain medications, less pain with intercourse	Left L1/S2 (2)	Migration of left S2 lead, but able to obtain adequate coverage with reprogramming	70	Yes, overall less pain, decreased pain medication, only remaining pain was deep vaginal pain	Yes
17	Be able to go back to work, wear underwear, sit for longer	Bilateral L1/S2 (4)	Migration of right S2 lead, turned off during trial	0	No, felt no relief of rectal pain, had leg cramping and tingling in the groin with stimulation	No
18	Be able to sit, resume gardening, increase walking distance, 50% reduction in pain	Left S2/S3 (2)	None	0	No, felt sitting was easier and less dyschezia on some days, but overall inconsistent during trial period and challenging to decide if it was helpful	No
19	Be able to sit, increase socialization and volunteer work	Bilateral L1/S2 (4)	None	40	No, helped with burning pain but no relief of pressure or foreign body sensation	No
20	50% reduction in pain, resume social activities, sit for 60 min, less painful intercourse	Left L1/S2 (2)	Procedural pain despite 10-day trial precluding evaluation	30	No, procedural pain throughout, did not want to extend trial period	No
21	Sit for longer, 50% pain reduction, try to hike/kayak, leave the house	Right L1/S2 (2)	Increased pain, L1 lead turned off	60	Yes, was able to sit for longer and leave the house, most relief in vaginal and coccyx pain	Yes
22	50% reduction in pain	Left L1/S2 (2)	Post-procedural pain, baseline pain worsened	0	No, vaginal/rectal burning was worse during the trial	No
23	50% reduction in pain, sit for greater than 30 min	Left L1/S2 (2)	None	80	Yes, vaginal and groin pain were clinically improved	Yes
24	50% reduction in pain, improve comfort with sitting, driving and volunteering	Bilateral L1/S2 (4)	None	70	Yes, able to sit longer	Yes
25	50% reduction in pain, be able to sit and stand longer, less urinary frequency	Left L1/S2 (2)	Post-procedural pain	>50	Yes, able to stand 45 min and cook, increased ability to socialize, able to reduce pain medication use by 50%	Yes
26	50% reduction in pain, improve function and relationship with husband	Bilateral L1 (2)	None	>50	Yes, relief of abdominal pain, no relief of “deeper bladder pain”	Yes
27	50% reduction in pain, be able to ambulate	Left S2, Right S3 (2)	None	0	No pain relief or improvement in urinary symptoms, ambulation	No
28	50% reduction in pain,	Right L1/S2 (2)	None	20	No pain relief	No
29	50% reduction in pain, be able to sit longer and wear underwear	Bilateral L1/S2 (4)	None	80	Yes, able to sit >2 h, able to wear underwear	Yes
30	50% reduction in pain, reduce pain with sitting	Bilateral L1/S2 (4)	S2 leads migrated after 3 days, patient lost some coverage and reprogrammed to optimize L1 leads	75	Yes, 60% relief of vaginal pain, 90% relief of rectal pain, able to sit more comfortably	Yes
31	50% reduction in pain	Bilateral L1/S2 (4)	None	60	Yes, pain on the left was 75–80% improved, on the right it was 60% improved, daily function improved	Yes

Table 2 summarizes the data collected from 31 patients trialed with a DRGS, including subjective data related to goals of therapy and relief, as well as objective data regarding the trial such as lead configuration and the number of leads, complications, and whether the patient proceeded to an implant.

Amongst patients with CPP caused by a surgical etiology, 14 out of 18 patients (78%, CI 54–92%) had a successful trial, as opposed to those with CPP caused by trauma (60%, CI 23–88%) or other causes (50%, CI 22–78%). Out of 31 patients, 29 had a pre-trial nerve block, most of which were pudendal nerve blocks, suggesting a diagnosis of pudendal neuralgia. Of the 29 who had a nerve block, 22 (76%, CI 58–88%) reported >50% reduction in pain for the duration of the local anesthetic.

Trial: The most common lead configurations utilized during DRGS trial were bilateral or unilateral L1 and S2 leads (21 out of 31 patients). Some notable exceptions are related to patient tolerance (ID Number 7) or insurance limitations (ID Number 2). The remaining lead configurations utilized for each patient's trial are presented in Table 2. Of the 31 patients who underwent DRGS trial, 21 patients (68%, CI 50–81%) reported >50% pain relief. Many patients had relief of some symptoms and not others, as reported in Table 2. There were patient, procedural, or systemic issues reported in 10 of the 31 patients during the trial period. Two patients experienced unanticipated skin reactions, one thought to be secondary to the adhesive and the other secondary to chlorhexidine. Four patients experienced lead migrations, all of which involved sacral leads. There was one insurance-related issue precluding bilateral lead placement during the trial period. Two patients experienced a worsening flare of chronic pain during and after the trial period, one patient had new groin pain resulting in one lead being turned off during the trial, and three patients reported severe procedural-related pain and could not assess pain relief during their DRGS trial (Table 2).

Implant: Twenty of the 21 patients with a successful DRGS trial underwent implantation, the details of which are presented in Table 3. One patient (ID Number 1) with a successful trial had improved pain relief persisting after the trial leads were removed, and therefore never pursued permanent implantation. A total of 68 individual leads were implanted. The average length of follow-up was 28.2 ± 17.3 months. Lead configurations used for permanent implant were the same as the trial configurations in the majority of patients (14 of 20 patients, 70%). Lead configurations were altered from the trial in six of the patients based on feedback and response to the trial. The most common lead configurations used for permanent implant were bilateral or unilateral leads placed at L1 and S2 (14 patients), with the remaining lead configurations utilized presented in Table 3.

**Table 3:** Summary of patient implant data.

ID	Lead Configuration (number)	Revision required (number of revisions)	Adverse Events	Implant % Relief	Able to Decrease Pain Medications?	Still Implanted?	Overall Satisfaction, General Impressions/Feedback	Follow-Up (Months)
2	Bilateral L1/S2 (4) (trial was left S2)	Yes (3)	Lead migration, radiculitis	50	Yes	Yes	Functional goals met, satisfied, better quality of life, “Wasn't even living before and now I am,” continued bowel/bladder incontinence, urinary frequency, not MRI compatible	48
3	Left L1/S2 (2) (same as trial)	Yes (1)	Lead migration	40	Yes	Yes	Overall satisfied, able to sit longer, less painful intercourse	32
4	Bilateral L1/S2 (4) (same as trial)	Yes (1)	Nonfunctional lead due to high impedance	60	Yes	Yes	All functional goals met; very satisfied	48
5	Bilateral L1/S2 (4) (same as trial)	Yes (1)	Infection	75–80% initially	Initially, not sustained	No	Initially 75–80% relief with goals met and decreased pain medications until revision surgery, never able to get good relief after with continued vaginal and rectal pain	48
6	Bilateral S2/S3 (4) (same as trial)	No	No	0	No	No	Developed worsening pain with stimulation, never got relief, persistent generator site pain	8
7	Bilateral S2/S3 (4) (trial was bilateral S2 + left L1; L1 not helpful)	No	No	60	Yes	Yes	Overall functional goals met, very satisfied	34
8	Right L1/S2, Left S2 (3) (trial was right L1/S2)	No	No	55	Yes	Yes	Reports implant process was more painful than anticipated, reports persistent sensitivity at generator site, feels it is helpful but not a cure, overall satisfied with continued functional improvement	20
9	Right L1/S2 (2) (same as trial)	Yes (1)	Implantable pulse generator pocket pain	95	Yes	Yes	Feels excellent relief and functional goals met, very satisfied, still with persistent discomfort at generator site	12
10	Bilateral T12/S2 (4) (trial was R T12/L1)	Yes (1)	Lead migration	50	No	No	50% relief of abdomen/groin pain for about 6 months until left T12 lead migration, never got relief of testicular pain, S2 caused leg stiffness that did not resolve, ongoing soreness at generator site	5
14	Bilateral L1/S2 (4) (same as trial)	No	No	40	Yes	No	Initially got 50% improvement in low back pain and some relief of inner thigh pain but no vaginal/clitoral pain relief, symptoms of persistent genital arousal disorder worsened without relief, developed new leg stiffness and discomfort with stimulation, not MRI compatible	48
15	Bilateral S2/S3 (4) (trial was unilateral S2/S3)	No	No	80	Initially, not sustained	Yes	Very satisfied for ∼2 years, burning rectal pain returned after treatment for newly diagnosed genitourinary cancer, nonresponsive to reprogramming, now very dissatisfied	25
16	Left L1/S2 (2) (same as trial)	No	Lead migration and implantable pulse generator pocket pain, not revised	40	No	No	Not enough relief, overall disappointed, some relief for 2 months, then waning efficacy and lack of coverage of deep vaginal pain, lead migrations at L1 and S2, new lower extremity pain with stimulation, and persistent pain at generator site with migration of generator superficially, not MRI compatible	29
21	Bilateral L1 and right S2 (3) (trial was right L1 and S2)	Yes (1)	Lower extremity spasms and pain with stimulation and lack of rectal coverage, right S2 revised and S3 added	0	Yes	No	Never got relief, lack of rectal coverage, new lower extremity pain and spasm with stimulation, symptoms got worse after revision, explant complicated by lead breakage and inability to retrieve small lead fragment, later removed by neurosurgery as not MRI compatible	48
23	Left L1/S2 (2) (same as trial)	No	No	100	Yes	Yes	Very satisfied. “I would totally recommend it to anybody. It's worth everything. It changed my life."	7
24	Bilateral L1/S2 (4) (same as trial)	No	No lead migration or fracture, but worse pain in bilateral medial thighs, occasional stabbing pain in inner groin	50	No	Yes	Satisfied, can drive/sit for up to 2 h, can volunteer for a few hours, still sensitive to tighter underpants/clothes.	29
25	Left L1/S2 (2) (same as trial)	No	Occasional episodes of right penile pain	50	Initially, not sustained	Yes	Satisfied, he generally is “relatively pain free" for 10–14 days. Then pain can flare for 5–6 days. Urinary frequency has decreased by >50% at nighttime. Standing and walking are still limited by pain.	4
26	Bilateral L1/S2 (4) (trial was bilateral L1)	Yes (1)	Lead fracture and migration	50	Yes	Yes	Very satisfied, functional goals met, “I would do the surgery every year if I had to.”	13
29	Bilateral L1/S2 (4) (same as trial)	Yes (5)	Lead fracture (multiple episodes) and migration, implantable pulse generator pocket pain	80–90% initially, now 30–50%	No	Yes	Satisfied, was able to wear underwear and engage in sexual activity, however recent S2 lead fractures have disrupted pain relief	61
30	Bilateral L1/S2 (4) (same as trial)	No	Lead fracture of L1 (not revised)	65	Yes	Yes	Satisfied, good relief for 1 year, found to have fractured L1 lead, using remaining 3 leads with overall 65% relief	38
31	Bilateral L1/S2 (4) (same as trial)	No	Low back pain and loss of pelvic pain relief	50	No	No	Dissatisfied	7

Table 3 summarizes the data collected from 20 patients implanted with DRGS after trial, including subjective data related to overall satisfaction and feedback, as well as objective data such as lead configuration, revisions required, adverse events, and follow-up interval.

The most common adverse event was lead migration or fracture, which affected 7 out of 20 patients (35%) and 19 out of 68 leads (28%). Nine of the 20 implanted patients underwent revision surgery. Seven of these patients underwent one revision surgery, while two patients underwent three and five revision surgeries, for a total of 15 individual revision surgeries performed. The reasons for the revision surgeries included: lead fracture or migration (n = 9), persistent pain at the implantable pulse generator site (n = 2), lead exposure and infection (n = 1), a non-functional high-impedance lead of unknown etiology (n = 1), and unwanted side effects (either pain or spasm) secondary to stimulation at the planned location (n = 2) (Table 3). Of note, some patients experienced adverse effects but did not undergo revision surgery including pain at the implantable pulse generator site (n = 1), lead migration (n = 1), and/or worse or new occasional pain (n = 3). From our 7 patients with lead migration and/or fracture, we calculated an average of 10.9 ± 14 months (range 0.5–36.1 months) to the first revision, generally widely distributed during the 28.2 ± 17.3 months follow-up.

Seven of the 20 implanted patients ultimately had the device explanted, with an average time to explant of 12.5 ± 3 months. Two of these patients reported never getting relief after implantation (ID Numbers 6 and 21), whereas the other 5 patients reported some degree of partial relief initially (ID Numbers 5, 10, 14, 16, and 31). Reasons reported for explanation included the following: 1) lack of coverage of painful regions (ID Numbers 10, 14, and 16); 2) worsening pain or unwanted pain with the use of stimulation (ID Numbers 6, 10, 14, 16, and 21); and 3) persistent pain at the generator site (ID Numbers 6, 10, and 16). One patient's explant was complicated by a fracture in the S2 lead which precluded its retrieval, ultimately resulting in another operation (ID Number 21).

Thirteen of the 20 implanted patients remain implanted at the date of last follow-up, with an average overall reported percent relief of 55 ± 15%. Of our patients with DRGS still implanted, 92% report continued satisfaction: they are either “satisfied” (54%) or “very satisfied” (39%) with the DRGS results. Furthermore, our average length of follow-up was over two years (28.2 ± 17.3 months). Two of the 13 patients still implanted reported persistent pain at the generator site. Lastly, four of the 20 patients who underwent permanent implantation reported frustration about the device not being MRI-compatible.

## 4 Discussion

The challenges of effectively treating CPP via emerging neuromodulation techniques are becoming increasingly clear. Such obstacles stem from the complexity of pelvic neuroanatomy, diagnostic uncertainty amongst patients with pelvic pain, and high rates of periprocedural complications. This retrospective chart review analyzes a sample of patients with CPP who were trialed with DRGS and subsequently implanted, and highlights the variable experiences of patients within domains such as relief, functionality, adverse events, and explantation in both the short- and longer-term. While ≥60% of our patients endorsed satisfaction with DRGS, some reported ineffective coverage of specific painful regions (clitoral, vaginal, testicular, and rectal pain), whereas others experienced unwanted stimulation to untargeted body parts (lower extremities and pelvis). This is not surprising given the innervation of the pelvis by both lumbar and sacral nerve roots.

Overall, our study demonstrates that ≥60% of patients reported successful reduction in pain after both trial (68%, CI 50–81%) and implant (60%, CI 47–85%), which is consistent with most studies reporting favorable outcomes of >50% pain reduction [15,22]. The majority of implanted patients reported ongoing relief at the date of last follow-up, stating they are either “satisfied” or “very satisfied” with DRGS. Additionally, our study had more than a two-year follow-up on average, whereas many of the previous studies focus on outcomes less than one year after implantation [22]. While our study lacks statistical power to detect factors predicting successful trial or implant, we may compare our patient characteristics to those in studies powered sufficiently. Hunter et al. (2019) distributed an online questionnaire to chronic pain specialists across 14 institutions, including 217 patients trialed with DRGS for a broad range of diagnoses (including six patients with CPP), and identified several factors associated with DRGS trial success [15]. The most important predictor of trial success was percentage of coverage during the trial, with other predictors including the use of at least two leads during the trial, a pain description involving less than three dermatomes, a pre-existing nerve injury, and post-surgical pain [15]. Like Hunter et al. (2019), we did notice a relationship between patients with a presumed post-surgical etiology for pain and having a successful trial: 78% (CI 54–92%) of patients with a surgical etiology of their pain reported a successful trial, compared to those with trauma (60%, CI 23–88%) or other causes (50%, CI 22–78%) of pain. This suggests that the relationship between post-surgical pelvic pain and successful pain relief with DRGS should be studied further so as to improve patient selection for this technology.

While our data demonstrate that patients are overall satisfied with their decision to pursue DRGS, approximately 32% of trialed patients and 70% of implanted patients experienced an adverse event. Such events ranged from a new pain complaint related to the device to lead fracture and/or migration, which affected 35% of implanted patients and 28% of implanted leads, respectively. These complications frequently resulted in at least one surgical revision. This rate of fracture and/or migration has been reported in other published studies [22,25]. Chapman et al. (2021) demonstrated that anchoring the DRGS leads significantly reduced lead migration but not fracture [22]. In our study, the use of anchors was not standardized, however, its utility in reducing complications warrants further investigation. Another recently reported approach is implantation via the paramedian, ipsilateral method for lead placement [26]. This approach involves parallel entry to the spinous process to facilitate lead placement and lateralization of the leads in the epidural space. Two small case studies concluded that the ipsilateral approach helps to avoid lead fracture from repetitive tension forces exerted from paraspinal muscle activation or entrapment within the fascial planes [21,27]. In the present study, a contralateral approach was used for both DRGS trial and implantation. Regardless, our ability to report on the rates of fracture and migration in 20 patients with more than two years of follow-up is an important contribution to the literature – it is likely applicable to all patients with DRGS and suggests the need not only for technical improvements but also for discussion of these unique risks during the consent process. Since lead migration and fracture are common complications of DRGS implantation frequently leading to reoperation [12], further large-scale studies are warranted in effort to reduce the financial and emotional implications of revision surgery.

While this current study aims to provide procedural and outcome transparency and a characterization of factors related to the success or failure of DRGS, we acknowledge several notable limitations. First, this is a retrospective chart review, and as such there was no standardization of patient selection, pain diagnoses, lead configuration, or follow-up intervals. Furthermore, the outcome measures used in this study rely solely on subjective, patient-reported experiences in pain reduction and satisfaction with patients' individual functional goals, thus subjecting our data to recall bias. Given the retrospective nature of the study, we were unable to obtain all of the implanted patients' pre- and post- NRS scores, and therefore we lack detailed categorical data on pain outcomes in the implanted patients, and can only comment on each patient's reported percentage relief. While our retrospective data enables us to provide descriptive information regarding the patient experience with DRGS implant, we are unable to make nuanced conclusions on the efficacy of this technology for pain relief. Future prospective studies using validated outcome measures such as the Visual Analog Scale/NRS, ODI, Patient-Reported Outcomes Measurement Information System-29 (PROMIS-29), or Short Form Health Survey (SF-12) will greatly enhance the rigor and reliability of such data. Lastly, the small sample size and demographics of our patient population limit the generalizability of our results. While our patient population was recruited from both academic and private practice settings, larger, prospective randomized controlled trials or meta-analyses are needed to further determine optimal patient selection criteria and patient factors that predict success or failure of DRGS for CPP.

## 5 **Conclusions**

This study presents one of the largest samples of patients with CPP trialed with DRGS with an average of two years of follow-up. The results highlight the variable experiences of these patients after DRGS trial/implant and the need for individualized care. The patient experiences highlight the challenges of targeting CPP using neuromodulation given the complex neuroanatomy and innervation of the pelvis (summary data presented in Table 4). While implanted patients experienced a 60% (CI 47–85%) success rate on average, we observed a high incidence of lead migration and fracture (35% of patients) and need for revision surgery (45% of patients). Perhaps more striking, given the frequency of migration and fracture, a total of 15 revision surgeries were performed on the 20 implanted patients and the explant rate was 35%, which is sparingly described in the literature.

**Table 4:** Summary of outcomes for DRGS trials and implants.

	Outcome
Most common lead configuration for Trial and Implant	Bilateral or unilateral L1/S2
Average % Pain Relief during Trial	48 ± 18% (n = 31)
Average % Pain Relief for Implanted Patients	55 ± 15% (n = 20)
Successful Trial (>50% relief)	68% (n = 31; CI 50–81%)
Successful Implant (>50% relief)	60% (n = 20, CI 47–85%)
Average Length of Follow-up for Implant	28.2 ± 17.3 months
Patient/procedural/systematic issues during Trial	32% (n = 31)
Patient/procedural/systematic issues during Implant	70% (n = 20)
Adverse events requiring revision surgery for Implanted Patients	45% (n = 20)
% Explanted	35% (n = 20)
Average time to Explant	12.5 ± 3 months

Table 4 presents summary outcome data from the 31 patients trialed and 20 patients implanted with DRGS.

DRGS may be an effective and life-changing therapy for patients suffering from CPP. However, the experience of our group and others demonstrate that patients implanted after a successful DRGS trial may experience waning pain relief over time, and potentially high rates of revision and/or explant. Patients should be counseled on the risk of DRGS complications involving lead migration or fracture and the potential need for revision surgery. Future studies should be performed to better understand which patients would most benefit from DRGS. Finally, studies to evaluate alternative techniques, such as the ipsilateral approach, are critical to help balance the benefit of this technology with current risks.

## Funding

This research did not receive any specific grant from funding agencies in the public, commercial, or not-for-profit sectors.

## Disclose

No financial support to disclose.

## Declaration of competing interest

The authors declare that they have no known competing financial interests or personal relationships that could have appeared to influence the work reported in this paper.
